# Supplementary material for: COUNTERING AGE-ASSOCIATED ALTERATIONS IN OLIGODENDROCYTE-DERIVED EXTRACELLULAR MATRIX REJUVENATES COGNITION
Source: bioRxiv. 2026 Feb 5:2026.02.03.702938. Preprint. [Version 1] doi: 10.64898/2026.02.03.702938 (PMC12889591; doi:10.64898/2026.02.03.702938)
Supplement: Supplementary file 3 [file media-3.pdf]

A)

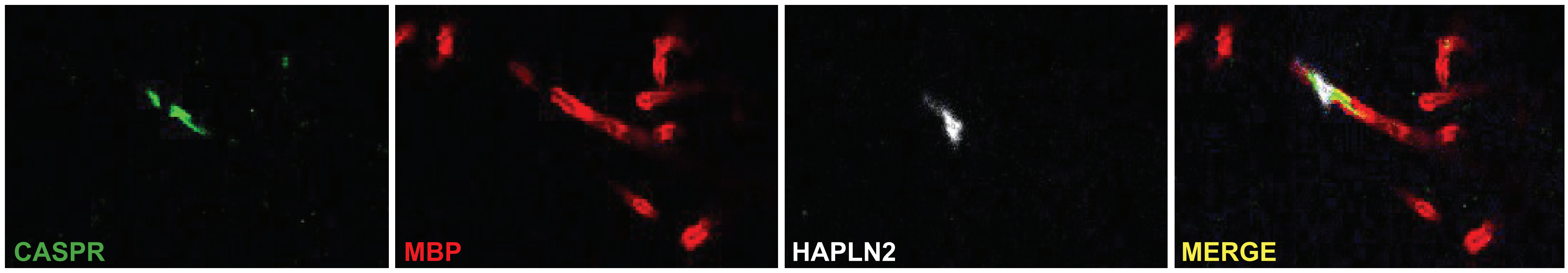

B)

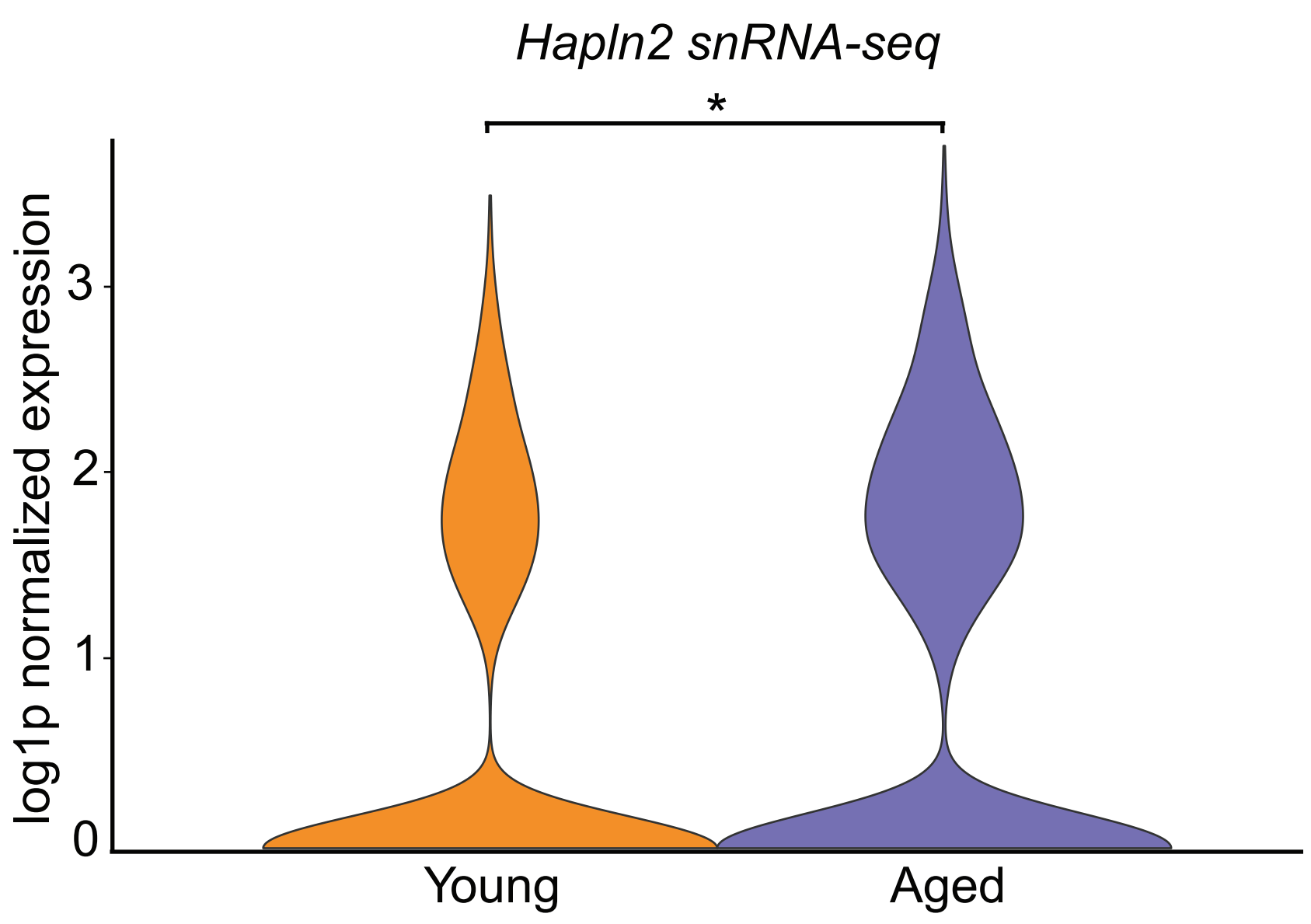

C)

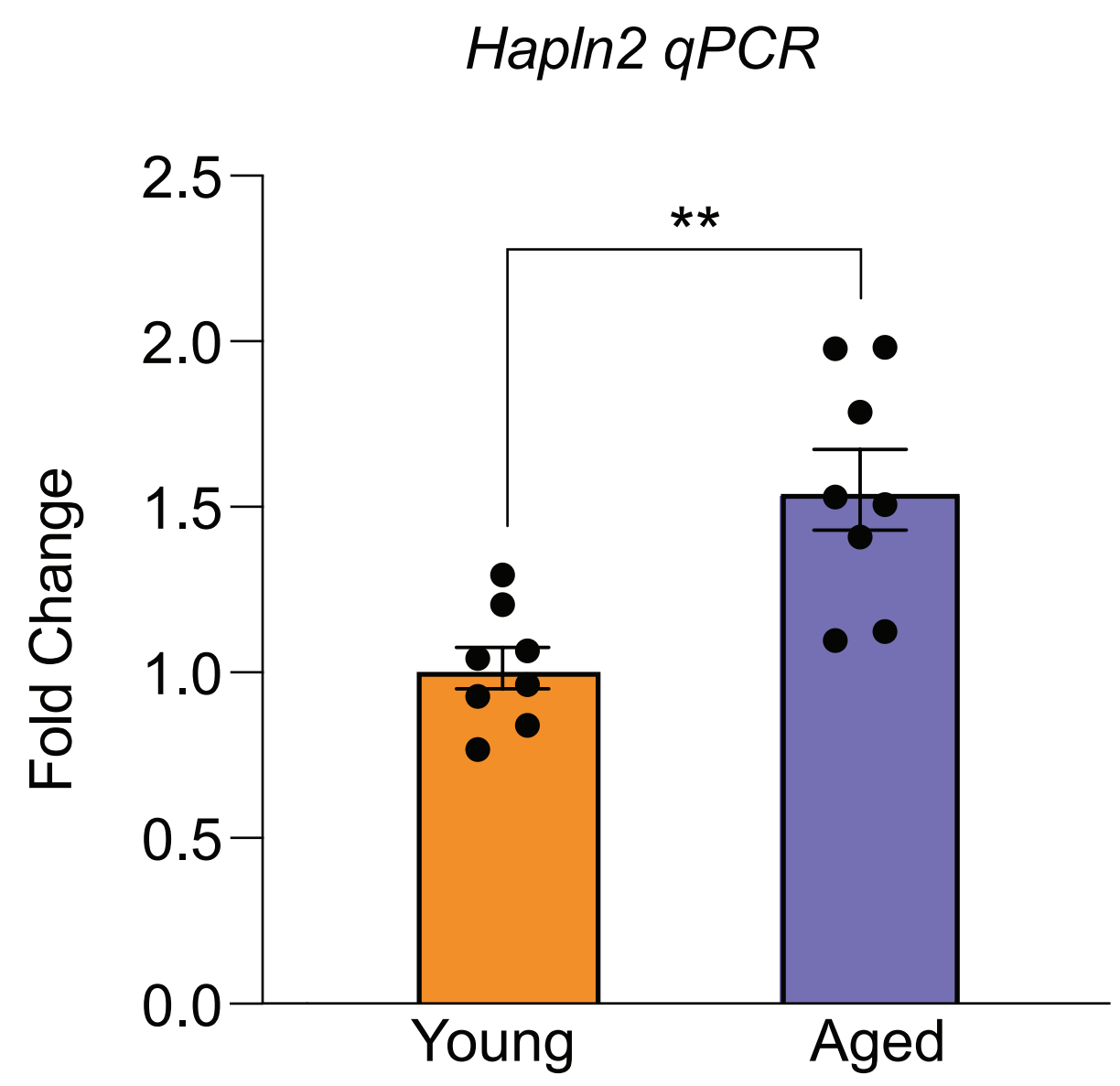

D)

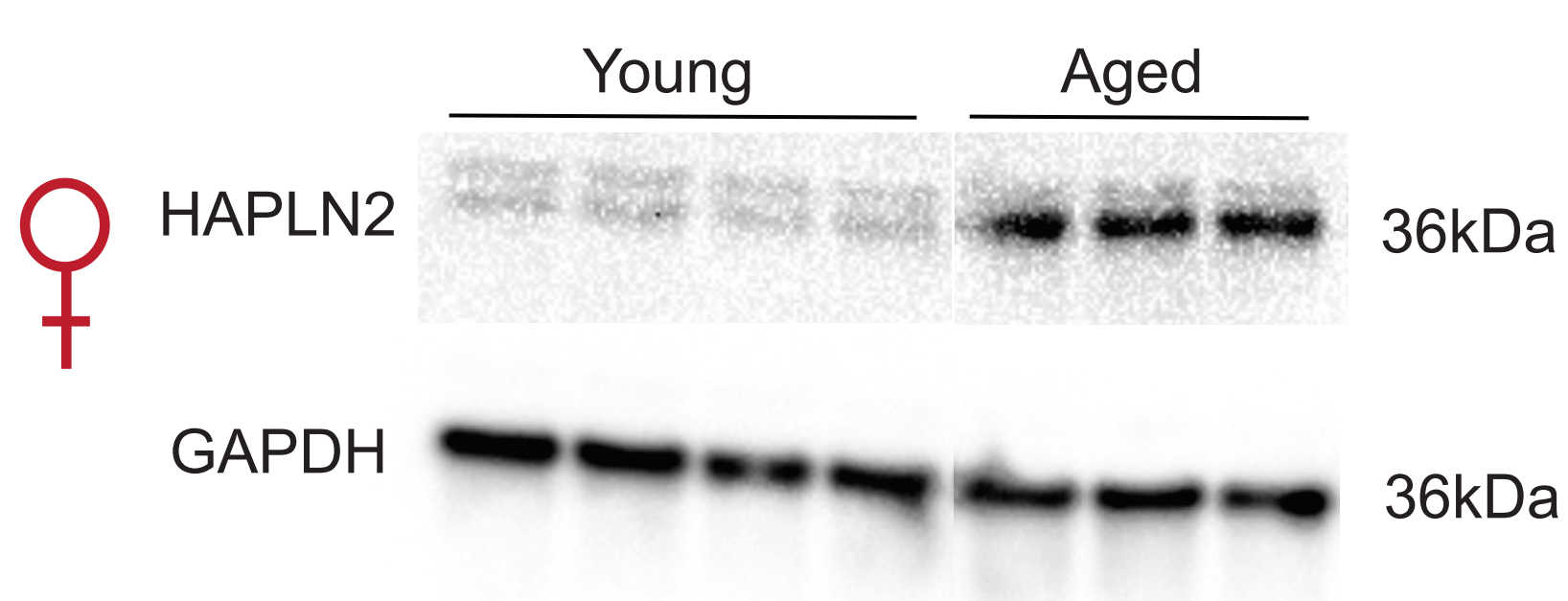

E)

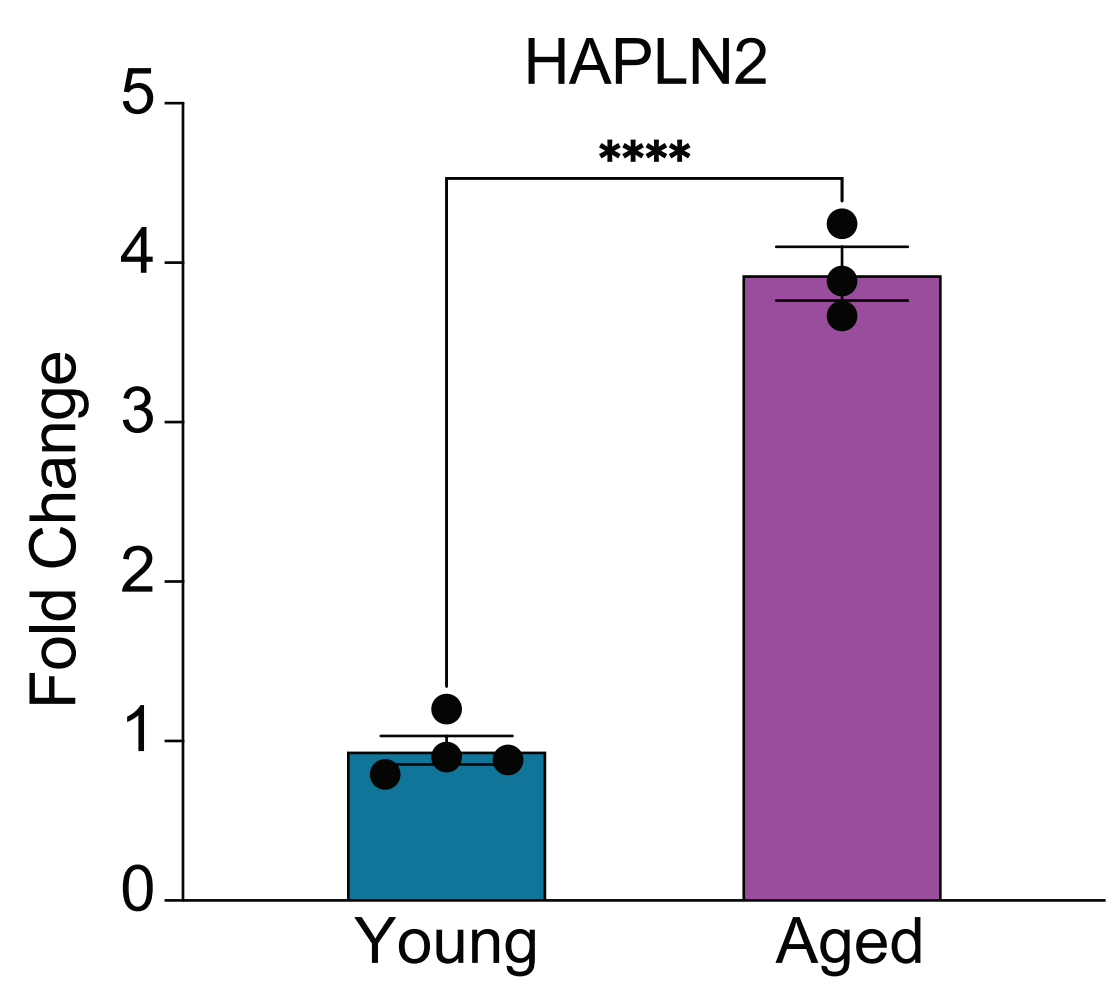

Supplementary Figure 3
